# Effect of Circadian Blood Pressure Variations on Retinal Microvascular Structures: Optical Coherence Tomography Angiography Analysis with the Nighttime Divided into Subintervals (Retinal Dawn Pattern)

**DOI:** 10.3390/medicina61101801

**Published:** 2025-10-06

**Authors:** Oğuzhan Zengin, Şule Nur Polat, Canan Satılmış, Burak Göre, Melike Yakut, İrem Aydoğmuş, Merve Çelik, Mehmet Önen, İhsan Ateş

**Affiliations:** 1 Department of Internal Medicine, University of Health Sciences, Ankara Bilkent City Hospital, Ankara 06800, Türkiye; sulenurpolat@outlook.com (Ş.N.P.); eskiturk.canan@gmail.com (C.S.); irem_aydogmus@hotmail.com (İ.A.); dr.ihsanates@hotmail.com (İ.A.); 2 Department of Internal Medicine, Çerkeş State Hospital, Çankırı 18600, Türkiye; 3 Department of Internal Medicine, Çifteler State Hospital, Eskişehir 26700, Türkiye; melikeyakut95@gmail.com; 4 Department of Internal Medicine, Gölbaşı Şehit Ahmet Özsoy State Hospital, Ankara 06830, Türkiye; merveevlii@gmail.com; 5 Department of Ophthalmology, Ankara Bilkent City Hospital, Ankara 06800, Türkiye; mehmetonen@hotmail.com

**Keywords:** tomography, optical coherence, angiography, blood pressure monitoring, ambulatory, circadian rhythm, hypertension, retinal vessels, capillaries

## Abstract

*Background and Objectives:* Circadian fluctuations in blood pressure, particularly the non-dipping pattern characterized by the absence of a nocturnal decline, are associated with an increased risk of microvascular complications. The retina, as a highly sensitive microvascular tissue, offers a valuable window into systemic hemodynamic alterations. However, the literature lacks detailed structural analyses that evaluate all retinal regions by segmenting nighttime into specific time intervals. Notably, the early morning period (04:00–08:00), during which stress hormones such as cortisol and catecholamines rise physiologically, leads to increased blood pressure that may significantly affect retinal microcirculation. This prospective study aims to assess retinal microvascular structures in dipper and non-dipper individuals using structural optical coherence tomography and to investigate their relationship with blood pressure parameters by dividing nighttime into distinct time segments. *Materials and Methods:* A total of 60 participants were classified as dipper (n = 26) or non-dipper (n = 34) based on 24 h ambulatory blood pressure monitoring results. Structural optical coherence tomography was used to evaluate superficial and deep capillary plexus densities in the foveal, parafoveal, and perifoveal regions, along with the area and perimeter of the foveal avascular zone (FAZ) and flow density (FD). Blood pressure values, including systolic, diastolic, mean arterial, and pulse pressure, were recorded during two nighttime intervals (00:00–04:00 and 04:00–08:00), and correlations with retinal parameters were analyzed. *Results:* No significant differences were observed in retinal microvascular parameters between the dipper and non-dipper groups. Deep capillary densities, particularly in the parafoveal and perifoveal regions, showed significant positive correlations with serum total protein, albumin, and very low-density lipoprotein (VLDL) levels. Furthermore, systolic and mean arterial pressures measured during the 04:00–08:00 interval demonstrated significant positive correlations with deep retinal vascular densities. The FAZ perimeter was negatively correlated with pulse pressure variability, while FD showed a negative correlation with mean arterial pressure variability. *Conclusions:* This prospective study is among the first to investigate the effects of circadian blood pressure patterns on retinal microvascular structures by segmenting nighttime into specific intervals and employing comprehensive structural optical coherence tomography across the entire retina. The findings suggest that retinal microvascular structure may be associated with fluctuations in blood pressure. Analyses of blood pressure measurements between 04:00 and 08:00 may offer supplementary insights into the evaluation of retinal microvascular structure.

## 1. Introduction

Hypertension stands as a leading global factor contributing to morbidity and mortality, affecting nearly one billion individuals globally [1]. Chronic elevation of blood pressure is a major contributing factor to severe long-term complications such as atherosclerotic cardiovascular disease, hypertensive retinopathy, cerebrovascular events, endothelial dysfunction, and glomerulosclerosis [2,3]. These complications are collectively referred to as Hypertension-Mediated Organ Damage (HMOD) [4]. Other metabolic disorders, including dyslipidemia and hyperglycemia, may also contribute to these pathological outcomes [5].

Circadian rhythm refers to physiological processes regulated by the body’s biological clock, occurring in cycles of approximately 24 h. Blood pressure is an important component of this rhythmic regulation [6]. In healthy individuals, blood pressure exhibits diurnal variation: it increases in the morning due to sympathetic nervous system activation and declines at night with the predominance of parasympathetic activity. This variation is classified into patterns such as “dipper” and “non-dipper” and is recognized as a key biomarker in cardiovascular risk assessment [7]. Disruption of this rhythm may predispose individuals to pathophysiological changes with prognostic relevance in hypertension and cardiovascular disease. For this reason, daily blood pressure distribution should be considered not only in diagnosis but also in treatment planning [8].

In optimal office measurements, a blood pressure below 120/70 mmHg is considered normal. Values between 120/70 and 140/90 mmHg indicate elevated blood pressure, whereas values ≥ 140/90 mmHg indicate hypertension. In ambulatory monitoring, a 24 h mean blood pressure of ≥130/80 mmHg is regarded as the diagnostic threshold for hypertension [4]. Under normal physiological conditions, nighttime blood pressure decreases by approximately 10–20% compared to daytime values, a phenomenon known as dipping. The absence of this decline defines the non-dipping pattern [5,9,10,11].

Several factors can impair this dipping pattern, including sodium and water retention, disturbances of circadian rhythm, and alterations in gut microbiota (dysbiosis) [12]. The non-dipper pattern is observed more frequently in older adults and in conditions such as obesity, diabetes mellitus, pheochromocytoma, Cushing’s syndrome, and renovascular hypertension [3,5]. Furthermore, studies have demonstrated that melatonin deficiency in non-dipper individuals may also contribute to the loss of the physiological nocturnal decline [13].

The earliest detectable effects of hypertension on the retina manifest as generalized narrowing of the retinal arterioles in response to elevated blood pressure [14]. Over time, intimal hyperplasia and hyaline degeneration occur, and compression of veins at arteriovenous crossing points may impair blood flow, leading to microaneurysm formation. These changes reflect irreversible retinopathy [15].

For hypertensive patients, particularly those with microvascular complications, diagnostic tools are needed to detect endothelial dysfunction and subclinical organ damage. The retina plays a pivotal role in the evaluation of systemic vascular disease because it is the only site where the microvasculature can be directly visualized non-invasively due to the transparency of ocular media [16]. Early detection of these changes in chronic hypertension is essential for preventing vision-threatening complications [17].

Optical coherence tomography angiography (OCT-A), a relatively recent imaging modality, offers a non-invasive, repeatable method that does not require contrast administration, making it a convenient option for patients [18,19,20]. OCT-A enables detailed quantitative evaluation of retinal capillary networks in both the deep and superficial plexuses, as well as parameters such as foveal avascular zone (FAZ) area, perifoveal and parafoveal capillary density. Image acquisition is based on the detection of light backscattering from moving erythrocytes within the vessels. This technology also facilitates the assessment of retinal microvascular rarefaction, enabling the early detection of hypertensive retinopathy [15,16,21,22,23].

In OCTA studies, significant retinal microvascular changes have been reported in patients with systemic hypertension. Especially in cases of long-term or uncontrolled hypertension, a decrease in vessel density has been observed in both the superficial and deep capillary plexuses. Additionally, changes reflecting an enlargement of the FAZ and alterations in vessel branching complexity have also been reported. The fact that these changes can be detected even before prominent signs of hypertensive retinopathy appear indicates that OCTA is a method capable of revealing subclinical microvascular damage. These findings suggest that OCTA can provide valuable information in revealing the microvascular consequences of hypertension and may serve as a useful, non-invasive tool for assessing systemic vascular health [24,25].

Despite multiple studies assessing the effect of hypertension on the retinal microvasculature, few studies have systematically examined the association between circadian blood pressure patterns and retinal vascular density using OCT-A data. In this regard, the main objective of the study was to investigate the effects of circadian blood pressure patterns (dipper vs. non-dipper), determined through 24 h ambulatory blood pressure monitoring, on retinal microvascular structures. As a secondary aim, we sought to investigate the potential associations between retinal vascular density—considered a marker of target organ damage—and blood pressure measurements obtained during specific nocturnal intervals (00:00–04:00 and 04:00–08:00). By addressing both circadian pattern differences and detailed nocturnal hemodynamic changes, this study represents one of the few prospective investigations to comprehensively assess their impact on retinal microvasculature.

## 2. Methods

### 2.1. Study Design and Population

This study was designed as a prospective, cross-sectional, and observational clinical investigation. The study population consisted of adult individuals who presented to the internal medicine outpatient clinic and met the predefined eligibility criteria.

### 2.2. Inclusion and Exclusion Criteria

The inclusion criterion was being over 18 years of age. Exclusion criteria included being under 18 years of age; a diagnosis of diabetes mellitus; retinal tumor; retinal detachment; congenital retinal anomalies; vitreoretinal interface disorders; refractive error greater than ±3 diopters; history of intraocular retinal surgery; age-related macular degeneration; inherited retinal dystrophy; glaucoma; retinal vascular diseases; hypertensive crisis; or secondary hypertension. None of the participants included in the study had a prior diagnosis of obstructive sleep apnea (OSA). Furthermore, clinical symptoms commonly used to assess OSA risk—such as habitual snoring, witnessed apneas, and excessive daytime sleepiness—were not reported by any of the participants

### 2.3. Parameters Assessed

The primary parameters evaluated in the study were as follows:

- Vessel density values of the superficial and deep vascular layers, obtained automatically from 6 mm fovea-centered optical coherence tomography angiography (OCT-A) images using the device’s built-in software. Foveal avascular zone (FAZ) area, FAZ perimeter, and fractal dimension measurements, automatically calculated from the same OCT-A images.
- Twenty-four-hour mean systolic, diastolic, and mean arterial pressure (MAP) values.
- Daytime and nighttime systolic, diastolic, and mean arterial pressure values.
- Daytime and nighttime pulse pressure values.
- Variance, standard deviation, and coefficient of variation for systolic, diastolic, and mean arterial pressure values.
- Differences between daytime and nighttime measurements, such as the percentage decrease in nighttime systolic pressure.
- Classification of participants as dipper or non-dipper.
- Analysis of nighttime measurements by dividing them into two subintervals: 00:00–04:00 and 04:00–08:00.
- Available results of blood biochemistry and complete blood count tests.

### 2.4. Retinal Imaging Protocol

Structural retinal and vascular density measurements were obtained using spectral-domain optical coherence tomography and optical coherence tomography angiography (Optovue, RTVue XR100-22, serial number 0092000203007264). All examinations were performed by the same ophthalmologist experienced in operating the device. During the imaging process, scans were performed centered on the fovea (6 × 6 mm, fovea-centered), and each eye was imaged in the superficial and deep retinal layers using the device’s standard AngioRetina mode. When motion or segmentation artifacts were detected, the measurement was repeated until an acceptable quality was achieved with a signal strength index (SSI) of ≥7/10. All participants in the study met this signal strength index requirement. During processing, only images meeting this quality threshold were included in the analysis. Segmentation errors were not manually corrected; all parameters were obtained through the device software’s built-in automated processing pipeline. During measurement, vessel density of the superficial and deep capillary plexuses, as well as the FAZ area, perimeter, and FD, were automatically calculated using the device’s integrated AngioAnalytics software. The same standard algorithm was applied to all participants.

### 2.5. Blood Pressure Measurement Protocol

Ambulatory blood pressure monitoring (ABPM) was performed using a Suntech Bravo 222B device. Measurements were automatically taken every 30 min over a 24 h period. At least 16 readings were obtained during the daytime and at least 6 readings during the nighttime. The cuff was placed on the participant’s non-dominant arm. Data were analyzed using the device’s dedicated software. Twenty-four-hour mean values were calculated from the hourly averages of daytime and nighttime measurements. Variance, standard deviation, and coefficient of variation were computed separately for systolic, diastolic, and mean arterial pressure. Participants with a >10% reduction in nighttime blood pressure were classified as dippers, while those with a reduction < 10% were classified as non-dippers. Nighttime readings were further analyzed by dividing them into two subintervals: 00:00–04:00 and 04:00–08:00.

In all participants, ambulatory blood pressure monitoring and OCT/OCT-A imaging were performed on the same day. Additionally, results of blood biochemistry and complete blood count obtained within one month prior to study enrollment were recorded.

### 2.6. Statistical Analysis

All statistical analyses were performed using IBM SPSS Statistics for Windows, Version 26.0, IBM Corp., Armonk, NY, USA. A priori power analysis was conducted with G*Power 3.1 to determine the required sample size. The distribution of continuous variables was examined using the Shapiro–Wilk test. Data with normal distribution were expressed as mean ± standard deviation, whereas those without normal distribution were presented as median and interquartile range. Categorical variables were summarized as frequencies and percentages. Comparisons of categorical variables such as sex, hypertension, and coronary artery disease status were carried out using the chi-square test. Continuous variables with normal distribution were compared using the independent samples *t*-test, and those without normal distribution were analyzed with the Mann–Whitney U test. Given that the majority of retinal OCT and OCT-A parameters did not follow a normal distribution, the Mann–Whitney U test was employed for their group comparisons. Correlations between retinal parameters and biochemical, hematological, and blood pressure variables were evaluated using Spearman’s rank correlation coefficient, and the strength and direction of the associations were interpreted accordingly. All statistical tests were two-tailed, with a *p*-value of less than 0.05 considered statistically significant and a *p*-value of less than 0.01 considered highly significant.

### 2.7. Ethics Statement

**Figure 1:**
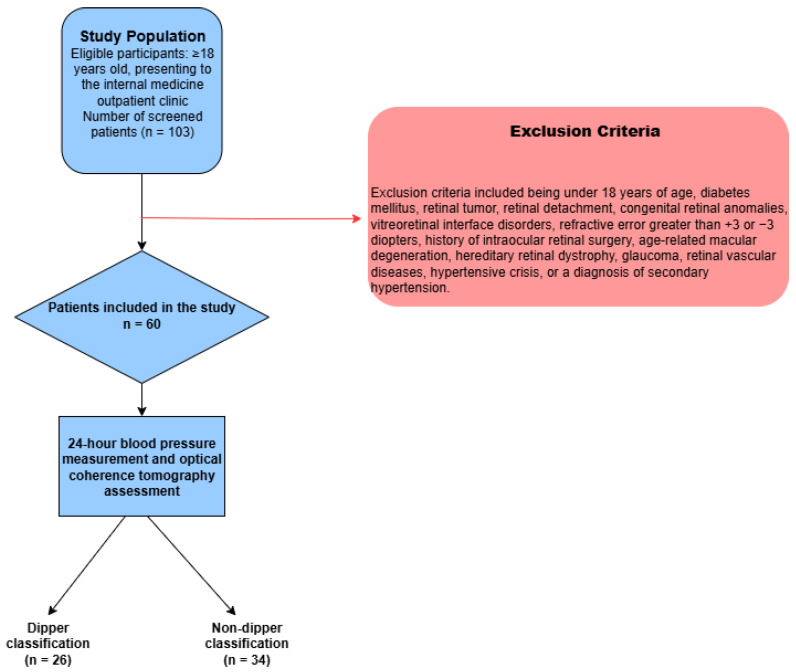
Flowchart of the study.

This study was approved by the Ethics Committee of Ankara Bilkent City Hospital on 30 April 2025, with decision number TABED 2-25-1113. The research was conducted in accordance with the principles of Good Clinical Practice and the Declaration of Helsinki, including its later amendments. Written informed consent was obtained from all participants prior to their inclusion in the study. The study design is presented in Figure 1.

## 3. Result

The demographic characteristics of the participants are presented in Table 1. Of the total 60 participants, 34 were classified as non-dippers and 26 as dippers. In the non-dipper group, 58.82% (n = 20) were female and 41.18% (n = 14) were male, whereas in the dipper group, 50.0% (n = 13) were female and 50.0% (n = 13) were male. The groups showed no significant differences in terms of sex distribution (*p* = 0.603). Similarly, no notable difference was detected regarding the presence of hypertension (*p* = 0.514). Hypertension was present in 47.06% (n = 16) of the non-dipper group and 50.0% (n = 13) of the dipper group. The mean age was 50.68 ± 12.73 years (median 48.50; IQR 22.00) in the non-dipper group and 44.85 ± 13.76 years (median 46.00; IQR 19.50) in the dipper group, without a meaningful statistical difference between the groups (*p* = 0.095).

Table 2 presents the comparison of retinal OCT parameters between dipper and non-dipper groups. Analysis showed no meaningful statistical differences between the groups in vessel density measurements of the deep and superficial capillary plexuses, including the whole image, fovea, parafovea, and perifovea regions, as well as their superior, inferior, nasal, and temporal quadrants (all *p* > 0.05). Similarly, analysis revealed no significant intergroup differences in the FAZ area, PERIM or FD values (*p* > 0.05). These findings indicate that, within the scope of this study, retinal microvascular parameters did not differ significantly based on the conventional dipper/non-dipper classification.

Table 3 summarizes the correlations between retinal OCT parameters and lipid profile as well as hematological markers. Spearman’s correlation analysis revealed multiple significant associations, particularly between deep capillary plexus vessel densities and lipid parameters. Deep capillary densities in several regions, including the whole image, superior and inferior hemispheres, parafoveal and perifoveal areas demonstrated significant positive correlations with VLDL cholesterol levels. Additionally, certain deep parafoveal and perifoveal segments were positively correlated with LDL cholesterol and non-HDL cholesterol.

Among hematological variables, no strong consistent associations were observed; however, select deep perifoveal parameters showed negative correlations with lymphocyte counts. In the superficial capillary plexus, correlations were generally weaker, but several parafoveal and perifoveal sectors exhibited significant negative correlations with HDL cholesterol and lymphocyte counts, and positive correlations with VLDL cholesterol.

For morphological indices, the FAZ area and FD showed positive correlations with total cholesterol and VLDL cholesterol, whereas PERIM correlated positively with total cholesterol. These findings suggest that systemic lipid metabolism parameters, particularly atherogenic lipid fractions, may be linked to specific regional retinal microvascular densities.

**Table 4:** The relationship between fundus OCT parameters and blood pressure indices.

Variable	S Systolic Variance	Systolic Standard Deviation	Systolic Coefficient of Variation	Diastolic Variance	Diastolic Standard Deviation	MAP Variance	MAP Standard Deviation	MAP Coefficient of Variation
D. Deep Whole Image	0.586/−0.072	0.715/−0.049	0.657/0.059	0.812/0.032	0.814/0.031	0.846/−0.026	0.580/−0.074	0.915/−0.014
D. Deep Superior- Hemi	0.472/−0.096	0.539/−0.082	0.312/0.134	0.927/0.012	0.908/−0.015	0.826/−0.029	0.499/−0.09	0.863/0.023
D. Deep Inferior- Hemi	0.565/−0.076	0.356/−0.122	0.973/0.004	0.737/−0.045	0.832/−0.028	0.785/−0.036	0.304/−0.136	0.732/0.046
D. Deep Fovea	0.869/0.022	0.929/−0.012	0.809/−0.032	0.802/0.033	0.718/0.048	0.773/0.038	0.656/−0.059	0.641/0.062
D. Deep Parafovea	0.885/−0.019	0.311/−0.134	0.636/0.063	0.600/−0.07	0.776/−0.038	0.976/0.004	0.192/−0.172	0.701/0.051
D. Deep Parafovea–Superior-Hemi	0.720/−0.048	0.345/−0.125	0.565/0.076	0.729/−0.046	0.838/−0.027	0.992/−0.001	0.270/−0.146	0.978/0.004
D. Deep Parafovea–Inferior-Hemi	0.926/0.012	0.316/−0.133	0.709/0.05	0.528/−0.084	0.744/−0.043	0.922/0.013	0.158/−0.186	0.456/0.099
D. Deep Parafovea–Tempo	0.983/0.003	0.385/−0.115	0.850/0.025	0.645/−0.061	0.746/−0.043	0.943/0.009	0.200/−0.169	0.586/0.072
D. Deep Parafovea–Superior	0.614/−0.067	0.393/−0.113	0.447/0.101	0.771/−0.039	0.311/−0.134	0.859/−0.024	0.328/−0.13	0.937/0.011
D. Deep Parafovea–Nazal	0.903/0.016	0.319/−0.132	0.326/0.13	0.718/−0.048	0.897/0.017	0.682/0.055	0.249/−0.153	0.470/0.096
D. Deep Parafovea–Inferior	0.868/−0.022	0.282/−0.142	0.924/−0.013	0.430/−0.105	0.865/0.023	0.893/−0.018	0.146/−0.192	0.807/0.033
D. Deep Perifovea	0.365/−0.12	0.386/−0.115	0.745/0.043	0.815/−0.031	0.769/−0.039	0.614/−0.067	0.357/−0.122	0.815/0.031
D. Deep Perifovea - Superior - Hemi	0.306/−0.135	0.499/−0.09	0.389/0.114	0.981/0.003	0.662/−0.058	0.621/−0.066	0.521/−0.085	0.890/0.018
D. Deep Perifovea–Inferior–Hemi	0.472/−0.096	0.346/−0.125	0.925/−0.013	0.720/−0.048	0.923/−0.013	0.675/−0.056	0.298/−0.138	0.733/0.045
D. Deep Perifovea–Tempo	0.697/−0.052	0.670/−0.057	0.751/0.042	0.968/0.005	0.768/0.039	0.927/−0.012	0.665/−0.058	0.797/0.034
D. Deep Perifovea–Superior	0.277/−0.144	0.552/−0.079	0.252/0.152	0.981/0.003	0.552/−0.079	0.558/−0.078	0.588/−0.072	0.947/−0.009
D. Deep Perifovea–Nazal	0.523/−0.085	0.297/−0.138	0.992/−0.001	0.744/−0.043	0.872/−0.021	0.769/−0.039	0.228/−0.159	0.780/0.037
D. Deep Perifovea–Inferior	0.260/−0.149	0.276/−0.144	0.907/−0.016	0.579/−0.074	0.540/−0.081	0.429/−0.105	0.279/−0.143	0.951/0.008
D. Superficial Whole Imaqe	0.724/0.047	0.664/0.058	0.487/0.092	0.498/0.09	0.368/−0.119	0.659/0.059	0.475/0.095	0.368/0.119
D. Superficial Superior–Hemi	0.865/0.023	0.666/0.057	0.453/0.1	0.590/0.072	0.302/−0.137	0.867/0.022	0.476/0.095	0.572/0.075
D. Superficial Inferior-Hemi	0.623/0.065	0.670/0.057	0.528/0.084	0.456/0.099	0.420/−0.107	0.525/0.084	0.501/0.089	0.249/0.152
D. Superficial Fovea	0.733/−0.045	0.444/0.102	0.436/−0.103	0.544/0.081	0.559/−0.078	0.742/−0.044	0.412/0.109	0.845/−0.026
D. Superficial Parafovea	0.559/0.078	0.369/0.119	0.531/0.083	0.272/0.145	0.850/−0.025	0.523/0.085	0.324/0.131	0.158/0.186
D. Superficial Parafovea–Superior–Hemi	0.588/0.072	0.400/0.112	0.497/0.09	0.252/0.152	0.935/0.011	0.522/0.085	0.310/0.134	0.207/0.167
D. Superficial Parafovea–Inferior–Hem	0.508/0.088	0.379/0.117	0.620/0.066	0.286/0.141	0.853/−0.025	0.477/0.094	0.364/0.12	0.130/0.199
D. Superficial Parafovea–Tempo	0.700/0.051	0.421/0.107	0.851/−0.025	0.332/0.128	0.652/−0.06	0.712/0.049	0.450/0.1	0.150/0.19
D. Superficial Parafovea–Superior	0.458/0.098	0.247/0.153	0.333/0.128	0.208/0.166	0.393/−0.113	0.491/0.091	0.168/0.182	0.140/0.194
D. Superficial Parafovea–Nazal	0.597/0.07	0.633/0.063	0.210/0.166	0.414/0.108	0.888/0.019	0.516/0.086	0.602/0.069	0.133/0.198
D. Superficial Parafovea–Inferior	0.607/0.068	0.328/0.13	0.874/0.021	0.267/0.147	0.728/0.046	0.514/0.087	0.272/0.145	0.367/0.12
D. Superficial Perifovea	0.891/0.018	0.791/0.035	0.535/0.082	0.629/0.064	0.295/−0.139	0.792/0.035	0.538/0.082	0.416/0.108
D. Superficial Perifovea–Superior–Hemi	0.946/−0.009	0.859/0.024	0.473/0.095	0.808/0.032	0.223/−0.161	0.947/−0.009	0.593/0.071	0.609/0.068
D. Superficial Perifovea–Inferior–Hemi	0.725/0.047	0.689/0.053	0.585/0.073	0.417/0.108	0.435/−0.104	0.519/0.086	0.443/0.102	0.243/0.154
D. Superficial Perifovea–Tempo	0.894/0.018	0.327/0.13	0.899/−0.017	0.289/0.14	0.649/−0.061	0.774/0.038	0.226/0.16	0.351/0.124
D. Superficial Perifovea–Superior	0.804/−0.033	0.848/−0.025	0.270/0.146	0.792/−0.035	0.149/−0.19	0.635/−0.063	0.912/0.015	0.864/0.023
D. Superficial Perifovea–Nazal	0.675/0.056	0.835/0.028	0.543/0.081	0.559/0.078	0.477/−0.094	0.516/0.086	0.562/0.077	0.422/0.107
D. Superficial Perifovea–Inferior	0.643/0.062	0.806/0.033	0.372/0.118	0.470/0.096	0.388/−0.114	0.474/0.095	0.565/0.076	0.113/0.208
FAZ (Mm^2^)	0.526/0.084	0.979/−0.004	0.503/0.089	0.584/−0.073	0.663/−0.058	0.869/0.022	0.993/0.001	0.892/0.018
PERIM (Mm)	0.260/0.149	0.605/0.069	0.566/0.076	0.898/−0.017	0.957/0.007	0.638/0.062	0.622/0.065	0.830/0.029
FD	0.276/0.144	0.987/−0.002	0.707/0.05	0.567/0.076	0.896/0.017	0.226/0.16	0.950/−0.008	0.047/0.260 *

**Figure 2:**
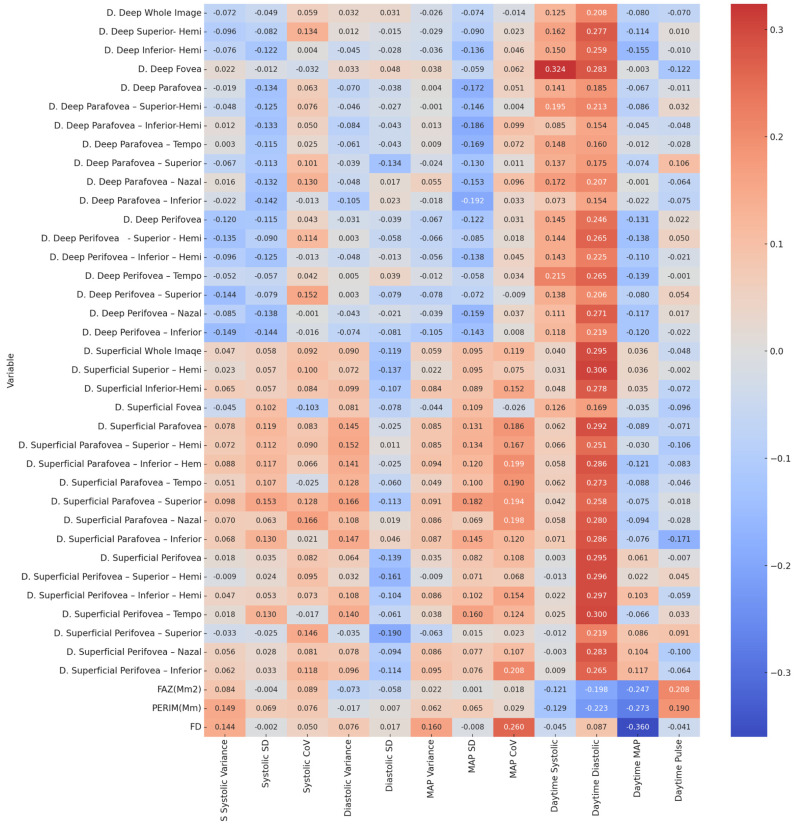
This figure is a heatmap illustrating the relationship between retinal OCT parameters and blood pressure variables. On the right side of the figure, there is a color scale indicating the strength of the Spearman correlation coefficient. Red tones represent positive correlations (becoming darker as the coefficient approaches +1), while blue tones represent negative correlations (becoming darker as the coefficient approaches −1). White or light tones indicate weak or no correlation (coefficient close to 0). Statistically significant correlations are denoted as follows: *p* < 0.01and *p* < 0.05. Abbreviations: FAZ: foveal avascular zone; PERIM: perimeter; FD: flow density; D: deep capillary plexus; S: superficial capillary plexus; SD: standard deviation; CoV: coefficient of variation. All values presented in the table represent Pearson correlation coefficients (r) between the variables.

The values in the table are presented in the format of *p*-value first, followed by the correlation coefficient (r) (*p*-value/*r*). Spearman’s correlation analysis was performed to evaluate the relationship between retinal OCT metrics and various blood pressure parameters, including systolic, diastolic, mean arterial pressure (MAP), pulse pressure, and their variabilities. Statistically significant correlations are denoted as follows: *p* < 0.05 (*), FAZ: foveal avascular zone, PERIM: perimeter, FD: flow density, D: deep capillary plexus. In the analysis, various significant correlations were observed between retinal optical coherence tomography parameters and blood pressure variables. Measurements of the deep and superficial capillary plexus showed predominantly positive correlations with daytime diastolic blood pressure, whereas more negative correlations were found with daytime mean arterial pressure. Certain regions, such as the deep fovea and superficial parafoveal segments, demonstrated moderate positive correlations with daytime systolic and diastolic pressures. In contrast, parameters such as the foveal avascular zone, perimeter, and fractal dimension were negatively associated with daytime mean arterial pressure, with the fractal dimension showing a particularly strong negative correlation. These findings suggest that specific retinal microvascular features are differently associated with various components of blood pressure, indicating potential structural and functional changes in retinal circulation related to hemodynamic alterations. The findings are shown in Figure 2.

Table 5 presents the correlations between retinal OCTA parameters and mean nighttime blood pressure values, segment-specific intervals (00:00–04:00 and 04:00–08:00), and the systolic change ratio. In the deep capillary plexus, mean nighttime MAP demonstrated multiple significant positive associations. The strongest correlations were observed with the deep superior-hemi (r = 0.426, *p* < 0.01), deep inferior-hemi (r = 0.343, *p* < 0.01), deep perifovea superior-hemi (r = 0.365, *p* < 0.01), and deep perifovea temporal (r = 0.391, *p* < 0.01) regions. During the 04:00–08:00 interval, systolic and MAP values were also positively related to several sectors, including the deep fovea, deep perifovea temporal, and deep perifovea superior subfields. In the superficial capillary plexus, fewer significant associations were observed. Notably, 04:00–08:00 systolic pressure correlated positively with the superficial parafovea superior-hemi (r = 0.282, *p* < 0.05) and superficial parafovea superior (r = 0.297, *p* < 0.05). FAZ, PERIM, and FD values showed no significant correlations with nighttime blood pressure or variability parameters. Taken together, these findings indicate that mean nighttime MAP and early morning systolic/MAP values are more strongly associated with deep retinal microvascular density, while the superficial plexus demonstrates weaker and more region-specific correlations.

Table 6 presents the correlations between retinal OCTA parameters and mean nighttime blood pressure values, including sub-interval analyses (00:00–04:00 and 04:00–08:00) and the systolic change ratio.

In the deep capillary plexus, nighttime MAP showed consistent positive correlations across several regions, with the strongest associations observed in the deep superior-hemi (r = 0.426, *p* < 0.01), deep inferior-hemi (r = 0.343, *p* < 0.01), deep perifovea superior-hemi (r = 0.365, *p* < 0.01), and deep perifovea temporal (r = 0.391, *p* < 0.01). During the 04:00–08:00 interval, systolic and MAP values were positively correlated with specific deep sectors, including the deep fovea, deep perifovea temporal, and deep perifovea superior, highlighting the early morning period as a critical window of vascular stress.

In the superficial capillary plexus, significant associations were less frequent. Notably, 04:00–08:00 systolic values correlated positively with the superficial parafovea superior-hemi (r = 0.282, *p* < 0.05) and superficial parafovea superior (r = 0.297, *p* < 0.05).

No significant relationships were detected between FAZ, PERIM, or FD and nighttime blood pressure parameters in this analysis. Overall, these findings suggest that mean nighttime MAP and early morning systolic/MAP values are more strongly associated with deep retinal microvascular density, whereas superficial plexus parameters show only limited correlations.

**Figure 3:**
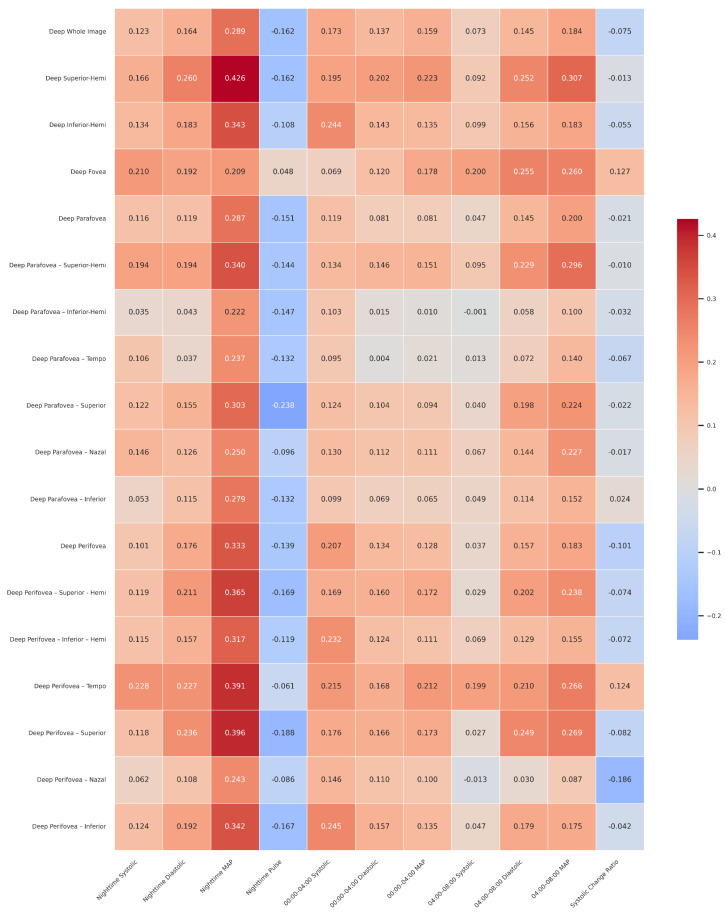
Heatmap showing the Spearman correlation coefficients between retinal deep capillary plexus optical coherence tomography measurements and blood pressure variability and mean values. Red tones represent positive correlations, and blue tones represent negative correlations. Statistically significant correlations are indicated with an asterisk. Abbreviations: MAP, mean arterial pressure; Systolic Change Ratio, systolic blood pressure change ratio; FAZ, foveal avascular zone; PERIM, perimeter; FD, flow density. All values presented in the table represent Pearson correlation coefficients (r) between the variables.

In this study, the relationship between retinal optical coherence tomography measurements and blood pressure parameters was evaluated. The analyzed variables included nighttime systolic, diastolic, mean arterial pressure, and pulse values, as well as systolic, diastolic, and mean arterial pressure measurements recorded during two separate nighttime intervals (00:00–04:00 and 04:00–08:00), and the systolic variability ratio. Spearman correlation analyses revealed statistically significant associations between certain retinal deep capillary plexus optical coherence tomography parameters and both blood pressure variability and mean values. A heatmap in Figure 3 illustrates these findings, with red indicating positive and blue indicating negative correlations.

As presented in Table 7, one of the most relevant ocular parameters, density deep in the superior-hemi region, was selected as the dependent variable for the regression analysis. The overall model was statistically significant (F = 5.537, *p* = 0.001), accounting for 43.7% of the variance (R^2^ = 0.437). Among the independent variables, only the 04:00–08:00 mean arterial pressure (MAP) showed a statistically significant association (B = 0.253, β = 0.604, *p* = 0.021), indicating a strong positive effect. This result suggests that higher early morning MAP values are linked with increased vessel density in the superior-hemi deep plexus. Other factors, including age, LDL cholesterol, non-HDL cholesterol, daytime and nighttime diastolic pressures, and nighttime MAP, were not statistically significant (*p* > 0.05). Since hypertension status and gender did not demonstrate meaningful associations with superior-hemi density in preliminary analyses, they were not entered into the regression model. Thus, the 04:00–08:00 MAP emerged as the most influential predictor in this analysis.

## 4. Discussion

In this prospective study, the effects of circadian blood pressure (BP) patterns on retinal microvascular structures were examined using optical coherence tomography angiography (OCT-A), with the nighttime period divided into two separate time intervals and evaluated across the entire retinal area. Our findings showed no statistically significant differences in retinal vascular parameters between dipper and non-dipper groups. However, systolic and mean arterial pressures measured particularly during the early morning hours (04:00–08:00) were positively correlated with deep capillary density. This result suggests that hemodynamic changes occurring during this physiologically active period, marked by a rise in stress hormones, may significantly influence retinal microcirculation.

Our study presents a novel contribution to literature by evaluating nighttime BP patterns not only based on the dipper/non-dipper classification but also across specific time intervals. This methodology extends beyond the traditional classification and offers an opportunity to explore the effects of early morning hemodynamic fluctuations on retinal microcirculation.

The absence of significant differences in demographic variables such as age, gender, and hypertension prevalence between the dipper and non-dipper groups indicates that the two groups shared similar baseline characteristics. This strengthens the validity of our findings, allowing for a more isolated evaluation of the physiological effects of circadian BP patterns.

In line with existing data suggesting an increased cardiovascular risk and target organ damage in non-dipper individuals [26], the potential impact on microcirculation has also drawn attention. However, previous studies have not considered dividing nighttime into specific intervals, as was the case in our study. Although various biomarkers have been investigated for early endothelial damage, there is still no definitive marker, which has led clinicians to focus on measurements indicating target organ involvement [27,28]. In our study, the effects on retinal microvascular structures were assessed in detail using optical coherence tomography and OCT-A. Nonetheless, no significant differences were found between dipper and non-dipper groups in either the superficial or deep retinal layers.

There are few studies examining the effects of circadian BP patterns on retinal microvasculature. In a noteworthy prospective study, Nolde et al. evaluated capillary vascular density using OCT-A in 142 hypertensive patients and found significantly lower vascular density in the foveal region of non-dipper individuals, suggesting a negative impact on retinal microcirculation due to insufficient nocturnal BP reduction [29]. Similarly, Nakano et al. reported more severe retinopathy and increased left ventricular hypertrophy in non-dipper hypertensive patients [9]. However, in our study, no statistically significant difference in retinal vascular density was observed between the two groups. Notably, our study included both hypertensive and normotensive individuals, distinguishing it from previous studies. The lack of a significant effect in the general population may suggest that the influence of the non-dipper pattern on retinal microvasculature may not be generalizable.

Furthermore, the subgroup analysis conducted within the hypertensive group also failed to reveal significant differences, indicating the necessity for larger-scale studies to verify these observations.

In a study assessing the relationship between nocturnal BP dipping and parapapillary choroidal vessel density in 267 normal-tension glaucoma patients, participants were divided into dipper, non-dipper, and over-dipper groups based on the magnitude of nocturnal BP drop. The study found no significant difference in peripapillary choroidal vessel density between the non-dipper and dipper groups [30], consistent with our results. Similarly, a study involving 115 hypertensive patients comparing retinal nerve fiber layer, macula, and optic nerve head parameters found no significant differences between dipper and non-dipper groups, aligning with our study findings [31].

However, our study did show significant and positive correlations between nighttime mean arterial pressure and deep retinal capillary plexus regions, particularly in the superior-hemi, perifoveal, and parafoveal areas of the DCP. These correlations suggest that BP fluctuations can affect the retinal microvasculature. In contrast, correlations with the superficial capillary plexus were weaker, indicating that the superficial retinal layer may be less responsive to BP variability.

Additionally, BP values measured during the early morning hours (04:00–08:00) were significantly associated with certain retinal regions. One physiological explanation for this is the activation of the renin–angiotensin–aldosterone system and the sympathetic nervous system during this period, leading to a marked rise in BP [32]. The positive relationship observed between average BP levels during these hours, and retinal vessel density highlights a novel perspective in the literature. These findings suggest that future studies should consider dividing the nighttime period into specific time intervals rather than relying solely on dipper/non-dipper classifications, as current literature lacks consensus on this matter.

Correlations involving the superficial vascular layers were fewer and weaker. This may indicate that the superficial capillary network is less sensitive to BP changes than the deep capillary network. The significant correlations observed during early morning BP elevations suggest the potential for structural changes in retinal microcirculation and support the utility of BP measurements during this window to predict the risk of hypertensive retinopathy.

Although this topic has been scarcely addressed in the literature, it carries pathophysiological importance. A valuable study by Jiao et al. found a significant negative correlation between high BP variability (e.g., real average variability, standard deviation) and vessel density (VD) and perfusion density (PD) in hypertensive individuals, although parameters such as mean arterial pressure variance or pulse pressure were not assessed [33].

The observed sensitivity of retinal microvasculature to systemic BP fluctuations suggests that OCT imaging of the fundus could be used not only diagnostically but also as a screening tool for early target organ involvement.

In OCT-A–based studies, the significance of the superficial retinal vascular network in hypertension remains unclear. Our study differed from prior literature by not focusing solely on hypertension but evaluating the general population, thus contributing uniquely to this area [16,34]. Indeed, studies investigating how retinopathy is affected in hypertensive patients with normal BP monitoring are limited. Therefore, we recommend prospective studies involving newly diagnosed hypertensive individuals and those with long-standing regulated BP.

A study by Carollo et al., investigating the potential relationship between retinal vascular density and renal resistive index, found reduced deep foveal plexus density in individuals with high renal resistive indices [11]. Additionally, those in the >75th percentile for resistive index had higher triglyceride levels and lower glomerular filtration rates. Consistent with this, our study revealed significant correlations between OCT-A parameters and biochemical variables such as total protein, albumin, very-low-density lipoprotein (VLDL), cholesterol, and urea demonstrating that systemic physiological status can significantly influence retinal microcirculation.

We found significant positive correlations between low-density lipoprotein (LDL), very-low-density lipoprotein (VLDL), total cholesterol levels, and OCT-A parameters. In particular, VLDL and cholesterol were positively correlated with DCP densities in parafoveal and perifoveal regions. These findings differ partially from some reports in the literature. For example, one study reported a negative correlation between high LDL-C levels and retinal vessel density [35]. This discrepancy may be explained by the younger and systemically healthier population in our study, the use of detailed retinal segmentation, and the combined analysis of various biochemical markers.

Additionally, compensatory adaptation in early stages may play a role. Elevated lipid levels may initially induce a transient increase in capillary density to maintain retinal perfusion, particularly in individuals without established vascular damage. Since none of our patients had a prior diagnosis of hyperlipidemia, subgroup analysis was not feasible in this study.

Recent studies have also shown that not only the retina, but also systemic microvascular structures are functionally affected by hypertension and age [36]. In a study examining cutaneous microcirculation using OCT-A during reactive hyperemia, individuals with high systolic BP demonstrated a diminished vascular response. Compared to younger individuals, older and hypertensive patients showed reductions in vessel density, vascular network length, and the number of branching points. These findings suggest that elevated BP is associated not only with structural changes but also with impaired microvascular function.

In this context, the relationship between circadian BP patterns and retinal microvascular parameters observed in our study is consistent with findings reported in other organ systems. A cross-sectional observational study involving 57 patients with a history of chronic hypertension but without signs of hypertensive retinopathy and 40 healthy volunteers demonstrated that even in the absence of clinical retinopathy, retinal microvascular and neural layers could be affected to a detectable degree using OCT-A [37]. Unlike that study, we analyzed both superficial and deep vascular structures across the entire retina and evaluated their relationship with subdivided nighttime periods and finding significant associations.

Circadian variations in blood pressure have been suggested as a contributing factor in the development and progression of optic nerve disorders such as glaucoma. In a study by Choi and Kook, increased nocturnal hypotension was found to be associated with primary open-angle glaucoma. Distinct from that work, our study assessed nighttime blood pressure by dividing it into specific time intervals, aiming to more precisely capture the influence of early morning rises in stress hormones—particularly cortisol and catecholamines—on blood pressure levels [38].

The association observed between higher systolic and mean arterial pressures during the early morning hours (04:00–08:00) and increased density of the deep retinal capillary plexus may have clinical implications not only for retinal microcirculation but also for optic nerve integrity. It is known that during this period, physiological surges in stress hormones can lead to elevated blood pressure, which may contribute to the pathogenesis of optic nerve conditions such as glaucoma and ischemic optic neuropathy. In glaucoma, circadian fluctuations in perfusion pressure can disturb the hemodynamic balance at the optic nerve head, potentially accelerating disease progression. Similarly, in ischemic optic neuropathy, elevated morning blood pressure may heighten microvascular stress and increase the risk of ischemic injury. Although our study did not specifically investigate glaucoma, by examining the effects of circadian hemodynamic changes on retinal microvasculature, it may provide indirect but meaningful insights into the early pathophysiological processes underlying optic nerve diseases. These findings may serve as a basis for future longitudinal research in this field [39,40,41].

The relationship identified in this study may serve as an early clinical marker for both ophthalmologists and clinicians involved in hypertension management. From a broader perspective, these findings highlight the importance of examining blood pressure variability during morning hours, beyond the traditional dipper/non-dipper classification, when assessing retinal microvascular health and optic nerve function. Prospective longitudinal studies are warranted to determine whether targeted blood pressure control during this critical time period exerts a neuroprotective effect on the optic nerve.

The circadian variations observed in our study may reflect the limits of retinal blood flow autoregulation. Under normal conditions, retinal vessels maintain stable perfusion despite fluctuations in systemic blood pressure through myogenic, endothelial, and metabolic mechanisms. However, it is known that this autoregulatory capacity can be impaired in the presence of chronic hypertension and other vascular risk factors. In our findings, the stronger associations between deep capillary plexus density and early morning systolic and mean arterial pressure suggest that the increase in blood pressure during this period may exceed the buffering capacity of autoregulation. Additionally, this mechanism is suggested to contribute to the pathophysiology of diseases involving the optic nerve and retinal vasculature, such as glaucoma, ischemic optic neuropathy, and diabetic retinopathy. Further studies are needed to explore this possibility [42,43,44,45].

This study has several limitations. Firstly, being conducted at a single center with a relatively small sample size and the exclusion of conditions such as diabetes may limit the generalizability of the findings. However, the fact that the study center functions as a referral institution receiving patients from various regions enhances the representativeness of the sample. Moreover, the exclusion of systemic conditions like diabetes, which can independently affect the retinal microvasculature, strengthens the internal validity of the study by allowing a more isolated assessment of the relationship between circadian blood pressure patterns and retinal microvascular parameters. Second, the cross-sectional design prevents causal inference between circadian BP patterns and retinal microvascular alterations. Third, although OCT-A provides high-resolution, non-invasive retinal imaging, segmentation errors and image quality variability may affect measurement accuracy. Fourth, BP measurements were based on 24 h ambulatory monitoring over a single day, precluding evaluation of inter-day circadian variability. Fifth, while potential confounders such as diabetes, ocular pathologies, and secondary hypertension were excluded, unmeasured factors such as lifestyle habits and subclinical systemic conditions may still influence the results. Additionally, lack of documentation on antihypertensive or other systemic medications constitutes a limitation, as these drugs may directly affect both BP patterns and retinal parameters. Nonetheless, since our primary aim was to explore the relationship between circadian rhythm and retinal microcirculation, this does not undermine the study’s core objectives. OSA may be a confounding factor in blood pressure variability. None of the participants had a prior diagnosis of OSA, and symptoms indicative of OSA were not observed. However, polysomnography, the gold-standard diagnostic method, was not performed; this represents a limitation of the study.

This study introduces a novel methodological approach to literature by segmenting the nighttime period into subintervals when evaluating the effects of circadian BP patterns on retinal microvascular structures. Our findings suggest that early morning hemodynamic fluctuations may significantly impact deep capillary plexus density. Although no significant differences were observed between dipper and non-dipper groups overall, subinterval analysis appeared to be more sensitive in detecting microvascular changes. Thus, moving beyond traditional classifications, this approach may offer added value in detecting early target organ damage and guiding individualized antihypertensive treatment strategies. However, longitudinal studies are needed to better clarify the causal nature of the observed associations and to assess potential microvascular changes over time.

## 5. Conclusions

In this prospective study, the effects of circadian blood pressure patterns on retinal microvascular structures were investigated through detailed optical coherence tomography angiography (OCT-A) analysis across the entire retina, with the nighttime period divided into specific time intervals. Although no statistically significant differences in retinal vascular parameters were observed between the dipper and non-dipper groups, certain metabolic and hemodynamic factors, such as hemoglobin, total protein, albumin, and VLDL levels, were found to be significantly associated, particularly with deep capillary density.

Moreover, a positive correlation between systolic and mean arterial pressures measured in the early morning hours (04:00–08:00) and deep retinal vascular density suggests that blood pressure elevations during this physiologically critical period may have potential implications for microvascular health.

This methodology goes beyond the traditional dipper/non-dipper classification by allowing an assessment of the possible effects of hemodynamic fluctuations, particularly those occurring in the early morning, on retinal microcirculation.

These findings suggest that the conventional dipper/non-dipper classification alone may be insufficient for fully assessing retinal risk in hypertensive individuals. Instead, segmenting nocturnal blood pressure measurements by time intervals, especially to include the early morning hours, may enhance the sensitivity in detecting microvascular alterations.

## Figures and Tables

**Table 1:** Demographic characteristics of participants according to blood pressure patterns.

Variable	Category	Blood Pressure				*p*
		Non-Dipper (n = 34)		Dipper (n = 26)		
		n	%	n	%	
Sex	Female	20	58.82	13	50.00	0.603
	Male	14	41.18	13	50.00	
Hypertension	No	18	52.94	13	50.00	0.514
	Yes	16	47.06	13	50.00	
**Variable**		**Mean ± SD**	**Median—IQR**	**Mean ± SD**	**Median—IQR**	***p***
Age (Years)		50.68 ± 12.73	48.50—22.00	44.85 ± 13.76	46.00—19.50	0.095

Categorical variables were compared using the chi-square test; continuous variables were analyzed using an independent sample *t*-test. A *p*-value less than 0.05 was considered statistically significant.

**Table 2:** Comparison of retinal OCT parameters between dipper and non-dipper groups.

Variable	Blood Pressure				*p*
	Non-Dipper (n = 34)		Dipper (n = 26)		
	Mean ± SS	Median —IQR	Mean ± SS	Median —IQR	
D. Deep Whole Image	44.66 ± 9.53	45.60—10.25	45.97 ± 4.20	46.30—6.48	0.720 ^u^
D. Deep Superior–Hemi	46.18 ± 5.72	46.50—9.12	46.13 ± 4.16	47.00—6.52	0.969 ^t^
D. Deep Inferior–Hemi	45.52 ± 6.72	45.30—11.33	45.77 ± 4.62	46.30—7.63	0.690 ^t^
D. Deep Fovea	34.15 ± 8.05	34.95—10.28	35.69 ± 8.05	34.00—11.2	0.466 ^t^
D. Deep Parafovea	52.24 ± 5.89	52.05—8.00	52.21 ± 3.69	51.65—6.82	0.822 ^t^
D. Deep Parafovea–Superior-Hemi	52.55 ± 5.75	52.25—6.95	52.25 ± 3.91	52.40—7.08	0.963 ^t^
D. Deep Parafovea–Inferior-Hemi	51.94 ± 6.29	51.85—8.42	52.15 ± 3.71	51.00—6.92	0.829 ^u^
D. Deep Parafovea–Tempo	53.31 ± 6.13	53.65—7.05	53.25 ± 3.66	53.95—5.68	0.972 ^t^
D. Deep Parafovea–Superior	51.74 ± 6.33	51.90—7.72	51.78 ± 3.63	51.00—6.17	0.884 ^t^
D. Deep Parafovea–Nazal	52.86 ± 6.05	52.65—9.1	52.65 ± 4.23	52.30—7.38	0.672 ^t^
D. Deep Parafovea–Inferior	51.09 ± 6.29	50.65—6.98	51.18 ± 4.72	49.45—8.05	0.835 ^u^
D. Deep Perifovea	46.83 ± 6.72	47.10—10.58	47.48 ± 4.57	48.60—7.57	0.768 ^t^
D. Deep Perifovea–Superior–Hemi	47.22 ± 6.11	48.00—9.68	47.64 ± 4.56	48.55—7.15	0.755 ^t^
D. Deep Perifovea–Inferior–Hemi	46.44 ± 7.75	45.75—12.52	46.98 ± 5.08	47.40—9.05	0.695 ^t^
D. Deep Perifovea–Tempo	50.85 ± 6.47	50.30—8.63	50.27 ± 4.39	50.50—7.43	0.576 ^t^
D. Deep Perifovea–Superior	45.80 ± 7.01	45.85—12.1	46.73 ± 5.36	47.65—9.92	0.978 ^t^
D. Deep Perifovea–Nazal	45.61 ± 6.53	44.60—11.10	45.57 ± 5.45	43.95—7.5	0.712 ^t^
D. Deep Perifovea–Inferior	45.22 ± 8.63	45.10—13.73	45.95 ± 5.69	47.15—10.22	0.928 ^t^
D. Superficial Whole Image	47.04 ± 4.51	48.20—7.40	47.13 ± 4.26	48.60—6.70	0.946 ^u^
D. Superficial Superior–Hemi	46.98 ± 4.42	47.70—7.73	47.24 ± 4.43	48.60—5.45	0.704 ^u^
D. Superficial Inferior-Hemi	47.11 ± 4.69	47.90—7.45	47.21 ± 4.14	48.50—4.95	0.712 ^t^
D. Superficial Fovea	19.80 ± 7.81	19.35—11.88	19.07 ± 8.62	17.15—7.00	0.928 ^t^
D. Superficial Parafovea	47.85 ± 5.94	49.10—9.57	48.19 ± 5.31	49.20—7.07	0.735 ^t^
D. Superficial Parafovea–Superior–Hemi	47.85 ± 5.97	49.10—10.52	47.36 ± 7.65	49.10—7.95	0.818 ^t^
D. Superficial Parafovea–Inferior–Hem	47.84 ± 6.13	49.45—9.15	48.12 ± 5.66	49.50—8.2	0.771 ^u^
D. Superficial Parafovea–Tempo	48.34 ± 5.33	49.00—7.9	48.80 ± 5.19	49.35—6.27	0.720 ^u^
D. Superficial Parafovea–Superior	48.21 ± 6.54	49.05—11.35	49.36 ± 5.28	50.75—5.27	0.779 ^t^
D. Superficial Parafovea–Nazal	45.61 ± 7.03	46.95—11.92	46.38 ± 6.19	47.05—9.63	0.469 ^t^
D. Superficial Parafovea–Inferior	49.02 ± 6.22	51.10—8.00	48.23 ± 6.11	49.60—7.20	0.658 ^t^
D. Superficial Perifovea	47.76 ± 4.57	48.85—6.65	48.27 ± 3.82	48.95—5.63	0.627 ^t^
D. Superficial Perifovea–Superior–Hemi	47.45 ± 4.46	47.70—7.58	48.12 ± 3.98	49.10—5.33	0.651 ^t^
D. Superficial Perifovea–Inferior–Hemi	47.95 ± 4.64	48.60—7.72	48.30 ± 3.87	49.20—6.28	0.547 ^t^
D. Superficial Perifovea–Tempo	43.15 ± 5.24	44.20—6.68	44.39 ± 4.44	44.50—7.17	0.300 ^u^
D. Superficial Perifovea–Superior	47.55 ± 4.52	46.80—7.95	48.66 ± 4.18	49.70—8.18	0.755 ^t^
D. Superficial Perifovea–Nazal	51.92 ± 4.30	52.40—8.48	51.30 ± 5.29	53.05—4.05	0.335 ^t^
D. Superficial Perifovea–Inferior	47.97 ± 5.01	48.05—6.68	48.47 ± 3.73	48.95—5.68	0.618 ^t^
FAZ (Mm^2^)	0.29 ± 0.08	0.29—0.11	0.30 ± 0.08	0.31—0.10	0.671 ^t^
PERIM (Mm)	2.08 ± 0.30	2.14—0.41	2.09 ± 0.30	2.14—0.38	0.578 ^t^
FD	51.24 ± 5.34	51.41—8.48	52.39 ± 5.43	52.98—6.79	0.927 ^t^

Comparison of retinal OCT parameters between dipper and non-dipper groups. Data are presented as mean ± standard deviation and median (interquartile range). Statistical comparisons were performed using an independent sample *t*-test (t) or Mann–Whitney U test (u), depending on data distribution. Abbreviations: D., vessel density; FAZ, foveal avascular zone; PERIM, perimeter of the foveal avascular zone; FD, flow density.

**Table 3:** Correlation between retinal OCT parameters and lipid profile and hematological markers.

Variable	Total Cholesterol (mg/dL)	Triglycerides (mg/dL)	HDL Cholesterol (mg/dL)	LDL Cholesterol (mg/dL)	VDL Cholesterol (mg/dL)	NON-HDL Cholesterol (mg/dL)	White Blood Cell Count (×10^3^/µL)	Neutrophil Count (×10^3^/µL)	Lymphocyte Count (×10^3^/µL)	Monocyte Count (×10^3^/µL)	Red Blood Cell Count (×10^6^/µL)	Red Cell Distribution Width (%)	Platelet Count (×10^3^/µL)	Hemoglobin (g/dL)
D. Deep Whole Image	0.080/0.228	0.386/0.114	0.798/0.034	0.051/0.253	0.203/0.167	0.028/0.283 *	0.182/−0.175	0.138/−0.194	0.451/−0.099	0.318/−0.131	0.939/0.01	0.836/0.027	0.078/−0.229	0.409/0.109
D. Deep Superior–Hemi	0.059/0.245	0.285/0.14	0.737/0.044	0.047/0.258 *	0.211/0.164	0.021/0.297 *	0.219/−0.161	0.124/−0.201	0.145/−0.19	0.213/−0.163	0.803/0.033	0.749/0.042	0.488/−0.091	0.328/0.129
D. Deep Inferior–Hemi	0.093/0.219	0.490/0.091	0.783/0.036	0.064/ 0.24	0.163/0.182	0.039/0.267 *	0.252/−0.15	0.183/−0.174	0.324/−0.129	0.069/−0.237	0.722/0.047	0.700/0.051	0.935/0.011	0.106/0.211
D. Deep Fovea	0.406/−0.109	0.566/−0.076	0.750/−0.042	0.443/−0.101	0.654/0.059	0.601/−0.069	0.692/−0.052	0.581/−0.073	0.367/−0.118	0.841/0.026	0.465/0.096	0.337/−0.126	0.990/−0.002	0.627/0.064
D. Deep Parafovea	0.103/0.213	0.288/0.139	0.354/0.122	0.060/0.244	0.079/0.228	0.029/0.281 *	0.289/−0.139	0.159/−0.184	0.712/−0.049	0.273/−0.144	0.946/−0.009	0.467/0.096	0.802/0.033	0.499/0.089
D. Deep Parafovea–Superior–Hemi	0.111/0.208	0.207/0.165	0.198/0.168	0.050/0.254	0.078/0.229	0.026/0.288 *	0.187/−0.173	0.097/−0.216	0.576/−0.074	0.256/−0.149	0.851/−0.025	0.561/0.077	0.843/−0.026	0.431/0.104
D. Deep Parafovea–Inferior–Hemi	0.109/0.209	0.401/0.11	0.584/0.072	0.084/0.225	0.095/0.217	0.042/0.263 *	0.437/−0.102	0.262/−0.147	0.870/−0.022	0.313/−0.133	0.960/0.007	0.406/0.109	0.507/0.087	0.598/0.07
D. Deep Parafovea–Tempo	0.039/0.268 *	0.339/0.125	0.753/ 0.042	0.026/0.288 *	0.100/0.215	0.010/0.329 *	0.305/−0.135	0.173/−0.178	0.960/−0.007	0.180/−0.176	0.930/0.012	0.481/0.093	0.679/0.055	0.432/0.103
D. Deep Parafovea–Superior	0.166/0.181	0.187/0.173	0.317/0.131	0.135/0.195	0.085/0.224	0.058/0.246	0.250/−0.151	0.148/−0.189	0.271/−0.144	0.165/−0.181	0.997/−0.001	0.708/0.049	0.494/−0.09	0.571/0.075
D. Deep Parafovea–Nazal	0.123/0.202	0.374/0.117	0.320/0.131	0.059/0.246	0.103/0.213	0.040/0.266 *	0.229/−0.158	0.096/−0.217	0.843/−0.026	0.298/−0.136	0.918/−0.014	0.420/0.106	0.917/−0.014	0.446/0.1
D. Deep Parafovea–Inferior	0.333/0.127	0.435/0.103	0.318/0.131	0.178/0.176	0.129/0.198	0.125/0.2	0.499/−0.089	0.363/−0.12	0.942/−0.01	0.681/−0.054	0.743/−0.043	0.384/0.114	0.206/0.165	0.844/0.026
D. Deep Perifovea	0.077/0.23	0.276/0.143	0.484/0.092	0.052/0.252	0.106/0.211	0.025/0.290 *	0.132/−0.197	0.086/−0.223	0.164/−0.182	0.082/−0.226	0.783/0.036	0.644/0.061	0.620/−0.065	0.076/0.231
D. Deep Perifovea–Superior–Hemi	0.031/0.278 *	0.224/0.159	0.585/ 0.072	0.050/0.255 *	0.145/0.19	0.016/0.309 *	0.152/−0.187	0.090/−0.221	0.059/−0.246	0.092/−0.219	0.560/0.077	0.660/0.058	0.418/−0.106	0.176/0.177
D. Deep Perifovea–Inferior–Hemi	0.164/0.182	0.362/0.12	0.432/0.103	0.060/0.144	0.106/0.211	0.041/0.265 *	0.174/−0.178	0.118/−0.204	0.408/−0.109	0.098/−0.215	0.983/−0.003	0.629/0.064	0.974/0.004	0.066/0.239
D. Deep Perifovea–Tempo	0.099/0.215	0.102/0.213	0.497/0.089	0.060/0.245	0.043/0.263 *	0.018/0.304 *	0.735/−0.045	0.509/−0.087	0.533/−0.082	0.200/−0.168	0.946/−0.009	0.572/0.074	0.691/0.052	0.275/0.143
D. Deep Perifovea–Superior	0.043/0.263 *	0.299/0.136	0.979/ 0.004	0.068/0.2637	0.305/0.135	0.025/0.289 *	0.147/−0.19	0.096/−0.217	0.022/−0.296 *	0.096/−0.217	0.432/0.103	0.782/0.036	0.270/−0.145	0.293/0.138
D. Deep Perifovea–Nazal	0.121/0.202	0.641/0.061	0.404/ 0.11	0.073/0.233	0.297/0.137	0.067/0.238	0.148/−0.189	0.101/−0.214	0.266/−0.146	0.025/−0.288 *	0.836/0.027	0.532/0.082	0.924/−0.013	0.064/0.241
D. Deep Perifovea–Inferior	0.263/0.147	0.468/0.096	0.304/0.135	0.105/0.111	0.149/0.189	0.092/0.219	0.104/−0.212	0.070/−0.236	0.390/−0.113	0.294/−0.138	0.923/−0.013	0.666/0.057	0.929/−0.012	0.097/0.216
D. Superficial Whole Image	0.271/0.144	0.939/0.01	0.079/0.228	0.641/0.161	0.601/−0.069	0.474/0.094	0.734/0.045	0.931/0.011	0.238/−0.155	0.060/−0.244	0.897/0.017	0.277/0.143	0.566/0.076	0.487/−0.092
D. Superficial Superior–Hemi	0.237/0.155	0.936/0.011	0.069/0.236	0.622/0.165	0.707/−0.05	0.408/0.109	0.731/0.045	0.921/0.013	0.328/−0.128	0.129/−0.198	0.911/0.015	0.248/0.151	0.546/0.08	0.452/−0.099
D. Superficial Inferior–Hemi	0.357/0.121	0.839/0.027	0.085/0.225	0.749/0.142	0.546/−0.079	0.605/0.068	0.783/0.036	0.945/0.009	0.149/−0.189	0.022/−0.295 *	0.858/0.024	0.330/0.128	0.760/0.04	0.627/−0.064
D. Superficial Fovea	0.054/−0.25	0.732/−0.045	0.066/−0.239	0.044/−0.261 *	0.947/0.009	0.111/−0.208	0.739/0.044	0.431/0.104	0.366/−0.119	0.080/−0.228	0.963/0.006	0.683/−0.054	0.780/−0.037	0.653/−0.059
D. Superficial Parafovea	0.121/0.203	0.792/0.035	0.034/0.274 *	0.722/0.047	0.982/−0.003	0.356/0.121	0.340/−0.125	0.282/−0.141	0.159/−0.184	0.015/−0.313 *	0.262/0.147	0.322/0.13	0.718/0.048	0.940/−0.01
D. Superficial Parafovea–Superior–Hemi	0.151/0.188	0.983/0.003	0.142/0.192	0.593/0.17	0.701/−0.051	0.397/0.111	0.565/−0.076	0.394/−0.112	0.374/−0.117	0.137/−0.194	0.480/0.093	0.346/0.124	0.261/0.147	0.604/−0.068
D. Superficial Parafovea–Inferior–Hem	0.117/0.205	0.636/0.062	0.024/0.290 *	0.649/0.06	0.792/0.035	0.276/0.143	0.393/−0.112	0.328/−0.129	0.172/−0.179	0.005/−0.361 **	0.243/0.153	0.361/0.12	0.775/0.038	0.926/−0.012
D. Superficial Parafovea–Tempo	0.020/0.300 *	0.934/0.011	0.022/0.294 *	0.451/0.0099	0.699/−0.051	0.198/0.168	0.551/−0.079	0.343/−0.125	0.355/−0.121	0.122/−0.202	0.041/0.264 *	0.396/0.111	0.367/0.118	0.862/−0.023
D. Superficial Parafovea–Superior	0.467/0.096	0.892/0.018	0.217/0.162	0.685/−0.005	0.642/−0.061	0.967/−0.005	0.409/−0.108	0.388/−0.114	0.132/−0.197	0.120/−0.203	0.711/0.049	0.226/0.159	0.660/0.058	0.712/−0.049
D. Superficial Parafovea–Nazal	0.081/0.227	0.664/0.057	0.032/0.277 *	0.465/ 0. 96	0.840/0.027	0.192/0.171	0.372/−0.117	0.283/−0.141	0.092/−0.22	0.005/−0.356 **	0.235/0.156	0.437/0.102	0.922/0.013	0.631/0.063
D. Superficial Parafovea–Inferior	0.310/0.133	0.454/0.099	0.023/0.293 *	0.905/0.016	0.595/0.07	0.421/0.106	0.220/−0.161	0.268/−0.145	0.259/−0.148	0.002/−0.395 **	0.465/0.096	0.355/0.122	0.985/−0.002	0.824/−0.029
D. Superficial Perifovea	0.262/0.147	0.941/0.01	0.130/0.197	0.497/0.089	0.720/−0.047	0.360/0.12	0.561/0.077	0.753/0.041	0.208/−0.165	0.062/−0.242	0.963/−0.006	0.303/0.135	0.747/0.042	0.765/−0.039
D. Superficial Perifovea–Superior–Hemi	0.164/0.182	0.716/0.048	0.087/0.223	0.465/0.096	0.871/−0.021	0.253/0.15	0.626/0.064	0.815/0.031	0.325/−0.129	0.105/−0.211	0.889/0.018	0.281/0.142	0.700/0.051	0.676/−0.055
D. Superficial Perifovea–Inferior–Hemi	0.472/0.095	0.857/0.024	0.203/0.167	0.637/0.062	0.597/−0.07	0.585/0.072	0.494/0.09	0.668/0.057	0.135/−0.195	0.043/−0.262 *	0.837/−0.027	0.312/0.133	0.721/0.047	0.851/−0.025
D. Superficial Perifovea–Tempo	0.572/0.074	0.731/0.045	0.127/0.199	0.755/0.0041	0.982/0.003	0.878/0.02	0.804/0.033	0.906/0.016	0.160/−0.184	0.470/−0.095	0.825/0.029	0.324/0.13	0.568/0.075	0.620/−0.065
D. Superficial Perifovea–Superior	0.092/0.219	0.721/0.047	0.073/0.233	0.260/0.148	0.990/−0.002	0.125/ 0.2	0.528/0.083	0.759/0.04	0.543/−0.08	0.117/−0.205	0.881/0.02	0.243/0.153	0.989/0.002	0.782/−0.036
D. Superficial Perifovea–Nazal	0.358/0.121	0.932/0.011	0.193/0.17	0.370/0.118	0.547/−0.079	0.353/0.122	0.561/0.077	0.735/0.045	0.155/−0.186	0.001/−0.417 **	0.894/−0.018	0.491/0.091	0.785/0.036	0.955/−0.007
D. Superficial Perifovea–Inferior	0.306/0.134	0.760/0.04	0.460/0.097	0.364/0.119	0.448/ −0.1	0.407/0.109	0.587/0.071	0.900/0.017	0.358/−0.121	0.269/−0.145	0.639/−0.062	0.310/0.133	0.449/0.1	0.895/−0.017
FAZ (Mm^2^)	0.025/0.289 *	0.898/0.017	0.771/0.038	0.099/0.215	0.724/0.046	0.083/0.226	0.696/0.052	0.875/0.021	0.497/0.089	0.651/0.06	0.392/0.113	0.538/0.081	0.992/−0.001	0.360/0.12
PERIM (Mm)	0.046/0.259 *	0.733/0.045	0.537/0.081	0.130/0.198	0.784/−0.036	0.183/0.174	0.609/0.067	0.801/0.033	0.240/0.154	0.055/0.249	0.626/0.064	0.551/0.079	0.499/0.089	0.953/−0.008
FD	0.013/0.319 *	0.308/0.134	0.557/0.077	0.111/0.208	0.633/0.063	0.036/0.271 *	0.716/0.048	0.753/−0.042	0.441/−0.101	0.085/−0.224	0.224/0.159	0.764/0.039	0.219/0.161	0.233/0.156

The values in the table are presented in the format of *p*-value first, followed by the correlation coefficient (r) *(p*-value/*r).* Spearman’s correlation analysis was used to examine associations between OCT measurements and lipid/hematologic parameters. Statistically significant correlations are indicated as follows: *p* < 0.01 (**) and *p* < 0.05 (*). FAZ: foveal avascular zone, PERIM: perimeter, FD: flow density. D: deep capillary plexus. Table 4 summarizes the correlations between retinal OCT parameters and blood pressure variability indices. In the deep capillary plexus, several significant associations were observed. Systolic and MAP variance demonstrated positive correlations with deep fovea (*p* = 0.012, r = +0.324) and deep inferior hemi (*p* = 0.045, r = +0.259). Additionally, diastolic variability showed weaker but consistent correlations across parafoveal and perifoveal regions. In the superficial capillary plexus, significant associations were less frequent but remained notable in parafoveal subfields, particularly between daytime diastolic values and superficial parafovea superior (*p* = 0.047, r = +0.258) and nasal regions (*p* = 0.030, r = +0.280). For morphological indices, FD showed a negative correlation with MAP coefficient of variation (*p* = 0.047, r = –0.260). No consistent associations were detected for FAZ or PERIM with blood pressure variability parameters.

**Table 5:** Relationship of Retinal OCT Metrics with Blood Pressure Variability and Mean Values.

Variable	00:00–04:00 Systolic	00:00–04:00 Diastolic	00:00–04:00 MAP	04:00–08:00 Systolic	04:00–08:00 Diastolic	04:00–08:00 MAP	Systolic Change Ratio
D. Deep Whole Image	0.187/0.173	0.297/0.137	0.226/0.159	0.581/0.073	0.274/0.145	0.162/0.184	0.571/−0.075
D. Deep Superior–Hemi	0.136/0.195	0.121/0.202	0.087/0.223	0.487/0.092	0.055/0.252	0.018/0.307 *	0.921/−0.013
D. Deep Inferior–Hemi	0.060/0.244	0.276/0.143	0.306/0.135	0.456/0.099	0.237/0.156	0.164/0.183	0.678/−0.055
D. Deep Fovea	0.602/0.069	0.361/0.12	0.174/0.178	0.128/0.2	0.052/0.255	0.047/0.260 *	0.334/0.127
D. Deep Parafovea	0.364/0.119	0.538/0.081	0.539/0.081	0.723/0.047	0.272/0.145	0.128/0.2	0.872/−0.021
D. Deep Parafovea–Superior–Hemi	0.309/0.134	0.267/0.146	0.251/0.151	0.474/0.095	0.081/0.229	0.023/0.296 *	0.941/−0.01
D. Deep Parafovea–Inferior–Hemi	0.435/0.103	0.907/0.015	0.937/0.01	0.994/−0.001	0.661/0.058	0.452/0.1	0.809/−0.032
D. Deep Parafovea–Tempo	0.472/0.095	0.976/0.004	0.873/0.021	0.924/0.013	0.587/0.072	0.291/0.14	0.613/−0.067
D. Deep Parafovea–Superior	0.343/0.124	0.428/0.104	0.475/0.094	0.766/0.04	0.132/0.198	0.088/0.224	0.867/−0.022
D. Deep Parafovea–Nazal	0.322/0.13	0.394/0.112	0.399/0.111	0.612/0.067	0.277/0.144	0.084/0.227	0.896/−0.017
D. Deep Parafovea–Inferior	0.450/0.099	0.600/0.069	0.620/0.065	0.711/0.049	0.390/0.114	0.252/0.152	0.857/0.024
D. Deep Perifovea	0.112/0.207	0.309/0.134	0.329/0.128	0.782/0.037	0.234/0.157	0.166/0.183	0.441/−0.101
D. Deep Perifovea–Superior–Hemi	0.198/0.169	0.221/0.16	0.189/0.172	0.828/0.029	0.126/0.202	0.070/0.238	0.577/−0.074
D. Deep Perifovea–Inferior–Hemi	0.074/0.232	0.347/0.124	0.398/0.111	0.605/0.069	0.332/0.129	0.241/0.155	0.586/−0.072
D. Deep Perifovea–Tempo	0.099/0.215	0.200/0.168	0.103/0.212	0.130/0.199	0.110/0.21	0.041/0.266 *	0.345/0.124
D. Deep Perifovea–Superior	0.180/0.176	0.205/0.166	0.185/0.173	0.837/0.027	0.057/0.249	0.039/0.269 *	0.532/−0.082
D. Deep Perifovea–Nazal	0.267/0.146	0.402/0.11	0.445/0.1	0.923/−0.013	0.822/0.03	0.510/0.087	0.154/−0.186
D. Deep Perifovea–Inferior	0.059/0.245	0.231/0.157	0.305/0.135	0.722/0.047	0.176/0.179	0.186/0.175	0.751/−0.042
D. Superficial Whole Image	0.213/0.163	0.724/0.046	0.252/0.15	0.059/0.247	0.327/0.13	0.298/0.138	0.970/−0.005
D. Superficial Superior–Hemi	0.210/0.164	0.837/0.027	0.304/0.135	0.052/0.255	0.319/0.132	0.254/0.151	0.877/−0.02
D. Superficial Inferior-Hemi	0.242/0.153	0.727/0.046	0.291/0.139	0.104/0.214	0.386/0.115	0.441/0.102	0.798/−0.034
D. Superficial Fovea	0.555/0.078	0.461/0.097	0.368/0.118	0.517/0.086	0.396/0.113	0.520/0.085	0.985/−0.002
D. Superficial Parafovea	0.369/0.118	0.930/0.012	0.361/0.12	0.110/0.21	0.664/0.058	0.615/0.067	0.642/−0.061
D. Superficial Parafovea–Superior–Hemi	0.377/0.116	0.651/0.06	0.166/0.181	0.030/0.282 *	0.436/0.103	0.233/0.158	0.490/0.091
D. Superficial Parafovea–Inferior–Hem	0.389/0.113	0.877/0.02	0.366/0.119	0.216/0.164	0.797/0.034	0.812/0.032	0.632/−0.063
D. Superficial Parafovea–Tempo	0.506/0.088	0.866/0.022	0.358/0.121	0.418/0.107	0.657/0.059	0.605/0.069	0.674/−0.055
D. Superficial Parafovea–Superior	0.724/0.047	0.931/−0.011	0.361/0.12	0.022/0.297 *	0.549/0.08	0.438/0.103	0.491/−0.091
D. Superficial Parafovea–Nazal	0.492/0.09	0.806/−0.032	0.637/0.062	0.339/0.127	0.820/0.03	0.847/0.026	0.459/−0.097
D. Superficial Parafovea–Inferior	0.457/0.098	0.627/0.064	0.241/0.154	0.056/0.25	0.732/0.046	0.688/0.053	0.885/−0.019
D. Superficial Perifovea	0.066/0.239	0.990/0.002	0.551/0.079	0.125/0.202	0.413/0.109	0.481/0.093	0.609/−0.067
D. Superficial Perifovea–Superior–Hemi	0.213/0.163	0.717/−0.048	0.754/0.041	0.158/0.186	0.573/0.075	0.580/0.074	0.477/−0.094
D. Superficial Perifovea–Inferior–Hemi	0.232/0.157	0.704/0.05	0.354/0.122	0.080/0.229	0.264/0.148	0.350/0.124	0.694/−0.052
D. Superficial Perifovea–Tempo	0.319/0.131	0.661/0.058	0.260/0.148	0.054/0.253	0.307/0.135	0.403/0.111	0.957/−0.007
D. Superficial Perifovea–Superior	0.224/0.159	0.539/−0.081	0.959/−0.007	0.346/0.125	0.666/0.057	0.702/0.051	0.236/−0.155
D. Superficial Perifovea–Nazal	0.320/0.131	0.903/−0.016	0.590/0.071	0.183/0.176	0.748/0.043	0.655/0.059	0.606/−0.068
D. Superficial Perifovea–Inferior	0.170/0.179	0.878/0.02	0.463/0.097	0.122/0.204	0.288/0.141	0.371/0.119	0.811/−0.032
FAZ (Mm^2^)	0.325/−0.129	0.554/−0.078	0.304/−0.135	0.170/−0.181	0.364/−0.12	0.230/−0.159	0.093/−0.219
PERIM (Mm)	0.551/−0.079	0.933/0.011	0.826/−0.029	0.460/−0.098	0.730/−0.046	0.755/−0.041	0.651/−0.06
FD	0.778/−0.037	0.316/−0.132	0.594/−0.07	0.899/−0.017	0.498/−0.09	0.408/−0.11	0.209/−0.165

The values in the table are presented in the format of *p*-value first, followed by the correlation coefficient (r) *(p*-value/*r*). *p* values were calculated using Spearman correlation test. * *p* < 0.05 indicates weak significant correlation. MAP: mean arterial pressure, FAZ: foveal avascular zone, PERIM: perimeter, FD: flow density, D: deep capillary plexus.

**Table 6:** Comparison of retinal OCTA parameters with daytime and nighttime blood pressure values.

Variable	Nighttime Systolic	Nighttime Diastolic	Nighttime MAP	Nighttime Pulse	Daytime Systolic	Daytime Diastolic	Daytime MAP	Daytime Pulse
D. Deep Whole Image	0.350/0.123	0.209/0.164	0.026/0.289 *	0.216/−0.162	0.344/0.125	0.111/0.208	0.546/−0.08	0.597/−0.07
D. Deep Superior–Hemi	0.204/0.166	0.045/0.260 *	0.001/0.426 **	0.215/−0.162	0.220/0.162	0.032/0.277 *	0.389/−0.114	0.937/0.01
D. Deep Inferior–Hemi	0.306/0.134	0.161/0.183	0.008/0.343 **	0.411/−0.108	0.257/0.15	0.045/0.259 *	0.240/−0.155	0.940/−0.01
D. Deep Fovea	0.108/0.21	0.141/0.192	0.112/0.209	0.717/0.048	0.012/0.324 *	0.029/0.283 *	0.979/−0.003	0.355/−0.122
D. Deep Parafovea	0.379/0.116	0.363/0.119	0.028/0.287 *	0.249/−0.151	0.286/0.141	0.156/0.185	0.614/−0.067	0.936/−0.011
D. Deep Parafovea–Superior–Hemi	0.137/0.194	0.137/0.194	0.008/0.340 **	0.272/−0.144	0.138/0.195	0.103/0.213	0.517/−0.086	0.807/0.032
D. Deep Parafovea–Inferior–Hemi	0.790/0.035	0.746/0.043	0.091/0.222	0.263/−0.147	0.522/0.085	0.241/0.154	0.735/−0.045	0.714/−0.048
D. Deep Parafovea–Tempo	0.421/0.106	0.779/0.037	0.071/0.237	0.315/−0.132	0.263/0.148	0.222/0.16	0.931/−0.012	0.834/−0.028
D. Deep Parafovea–Superior	0.354/0.122	0.238/0.155	0.020/0.303 *	0.067/−0.238	0.302/0.137	0.181/0.175	0.578/−0.074	0.420/0.106
D. Deep Parafovea–Nazal	0.265/0.146	0.338/0.126	0.056/0.25	0.465/−0.096	0.194/0.172	0.112/0.207	0.995/−0.001	0.629/−0.064
D. Deep Parafovea–Inferior	0.686/0.053	0.380/0.115	0.033/0.279 *	0.314/−0.132	0.582/0.073	0.240/0.154	0.872/−0.022	0.566/−0.075
D. Deep Perifovea	0.441/0.101	0.179/0.176	0.010/0.333 **	0.291/−0.139	0.273/0.145	0.058/0.246	0.324/−0.131	0.867/0.022
D. Deep Perifovea–Superior–Hemi	0.365/0.119	0.105/0.211	0.005/0.365 **	0.196/−0.169	0.275/0.144	0.041/0.265 *	0.296/−0.138	0.705/0.05
D. Deep Perifovea–Inferior–Hemi	0.382/0.115	0.231/0.157	0.014/0.317 *	0.363/−0.119	0.280/0.143	0.084/0.225	0.405/−0.11	0.875/−0.021
D. Deep Perifovea–Tempo	0.080/0.228	0.081/0.227	0.002/0.391 **	0.643/−0.061	0.102/0.215	0.041/0.265 *	0.294/−0.139	0.993/−0.001
D. Deep Perifovea–Superior	0.370/0.118	0.070/0.236	0.002/0.396 **	0.150/−0.188	0.296/0.138	0.114/0.206	0.546/−0.08	0.681/0.054
D. Deep Perifovea–Nazal	0.638/0.062	0.411/0.108	0.063/0.243	0.512/−0.086	0.401/0.111	0.036/0.271 *	0.378/−0.117	0.899/0.017
D. Deep Perifovea–Inferior	0.345/0.124	0.141/0.192	0.008/0.342 **	0.201/−0.167	0.374/0.118	0.093/0.219	0.367/−0.12	0.867/−0.022
D. Superficial Whole Imaqe	0.170/0.179	0.428/0.104	0.069/0.239	0.693/−0.052	0.764/0.04	0.022/0.295 *	0.788/0.036	0.717/−0.048
D. Superficial Superior–Hemi	0.179/0.176	0.447/0.1	0.052/0.254	0.738/−0.044	0.816/0.031	0.018/0.306 *	0.787/0.036	0.988/−0.002
D. Superficial Inferior–Hemi	0.236/0.155	0.502/0.088	0.143/0.193	0.780/−0.037	0.721/0.048	0.032/0.278 *	0.792/0.035	0.584/−0.072
D. Superficial Fovea	0.364/0.119	0.385/0.114	0.604/0.069	0.257/0.149	0.341/0.126	0.196/0.169	0.790/−0.035	0.466/−0.096
D. Superficial Parafovea	0.237/0.155	0.655/0.059	0.131/0.199	0.609/−0.067	0.643/0.062	0.024/0.292 *	0.504/−0.089	0.588/−0.071
D. Superficial Parafovea–Superior–Hemi	0.076/0.231	0.396/0.111	0.043/0.265 *	0.317/−0.131	0.619/0.066	0.053/0.251	0.819/−0.03	0.419/−0.106
D. Superficial Parafovea–Inferior–Hem	0.285/0.14	0.698/0.051	0.177/0.178	0.738/−0.044	0.662/0.058	0.027/0.286 *	0.359/−0.121	0.528/−0.083
D. Superficial Parafovea–Tempo	0.368/0.118	0.562/0.076	0.110/0.21	0.408/−0.109	0.638/0.062	0.035/0.273 *	0.508/−0.088	0.726/−0.046
D. Superficial Parafovea–Superior	0.242/0.153	0.723/0.047	0.090/0.222	0.307/−0.134	0.750/0.042	0.047/0.258 *	0.575/−0.075	0.894/−0.018
D. Superficial Parafovea–Nazal	0.531/0.083	0.854/0.024	0.204/0.168	0.868/−0.022	0.662/0.058	0.030/0.280 *	0.479/−0.094	0.834/−0.028
D. Superficial Parafovea–Inferior	0.089/0.221	0.581/0.073	0.273/0.145	0.994/0.001	0.594/0.071	0.027/0.286 *	0.568/−0.076	0.193/−0.171
D. Superficial Perifovea	0.417/0.107	0.597/0.07	0.150/0.19	0.754/−0.041	0.984/0.003	0.022/0.295 *	0.649/0.061	0.959/−0.007
D. Superficial Perifovea–Superior–Hemi	0.534/0.082	0.786/0.036	0.153/0.188	0.835/−0.027	0.924/−0.013	0.022/0.296 *	0.871/0.022	0.732/0.045
D. Superficial Perifovea–Inferior–Hemi	0.301/0.136	0.418/0.106	0.145/0.192	0.654/−0.059	0.866/0.022	0.021/0.297 *	0.436/0.103	0.655/−0.059
D. Superficial Perifovea–Tempo	0.443/0.101	0.300/0.136	0.073/0.235	0.912/−0.015	0.852/0.025	0.020/0.300 *	0.617/−0.066	0.803/0.033
D. Superficial Perifovea–Superior	0.686/0.053	0.996/−0.001	0.242/0.155	0.788/−0.036	0.925/−0.012	0.093/0.219	0.517/0.086	0.491/0.091
D. Superficial Perifovea–Nazal	0.298/0.136	0.894/0.018	0.358/0.122	0.648/−0.06	0.983/−0.003	0.028/0.283 *	0.433/0.104	0.447/−0.1
D. Superficial Perifovea–Inferior	0.380/0.115	0.546/0.08	0.173/0.18	0.538/−0.081	0.949/0.009	0.040/0.265 *	0.378/0.117	0.629/−0.064
FAZ (Mm^2^)	0.059/−0.245	0.544/−0.08	0.947/−0.009	0.931/−0.011	0.363/−0.121	0.128/−0.198	0.059/−0.247	0.110/0.208
PERIM (Mm)	0.180/−0.175	0.921/0.013	0.577/0.074	0.873/−0.021	0.329/−0.129	0.087/−0.223	0.037/−0.273 *	0.146/0.19
FD	0.408/−0.109	0.541/−0.08	0.326/0.13	0.706/−0.05	0.734/−0.045	0.510/0.087	0.005/−0.360 **	0.758/−0.041

The values in the table are presented in the format of *p*-value first, followed by the correlation coefficient (r) (*p*-value/*r*). Statistical analysis was performed using Spearman’s correlation test. ** *p* < 0.01 indicates strong significant correlation, while * *p* < 0.05 indicates weak significant correlation. MAP: mean arterial pressure; FAZ: foveal avascular zone; PERIM: perimeter; FD: flow density D: deep capillary plexus.

**Table 7:** Regression analysis results of variables influencing deep capillary plexus density in the superior hemisphere.

Variable	B	Sh	Beta	t	P	R^2^	F	p
Constant	21.181	5.675	−	3.732	**0.000**	0.437	5.537	**0.001**
Age (years)	−0.068	0.044	−0.178	−1.539	0.130			
LDL cholesterol (mg/dL)	0.050	0.039	0.310	1.282	0.206			
Non-HDL cholesterol (mg/dL)	0.010	0.035	0.071	0.293	0.770			
Daytime Diastolic BP (mmHg)	0.077	0.046	0.197	1.674	0.100			
Nighttime Diastolic BP (mmHg)	−0.165	0.128	−0.332	−1.286	0.204			
04:00–08:00 MAP (mmHg)	0.253	0.106	0.604	2.384	**0.021**			
Nighttime MAP (mmHg)	0.058	0.080	0.147	0.729	0.469			

Dependent variable: deep capillary plexus density in the superior hemisphere. LDL, low-density lipoprotein; MAP, mean arterial pressure; BP, blood pressure; Non-HDL, non-high-density lipoprotein. Statistically significant results are indicated in bold (*p* < 0.05).

## Data Availability

The original contributions presented in this study are included in the article. Further inquiries can be directed to the corresponding authors.

## Abbreviations

ABPM	Ambulatory Blood Pressure Monitoring
OCT	Optical Coherence Tomography
FAZ	Foveal Avascular Zone
PERIM	FAZ Perimeter
FD	Flow density
MAP	Mean Arterial Pressure
SBP	Systolic Blood Pressure
DBP	Diastolic Blood Pressure
PP	Pulse Pressure
SCP	Superficial Capillary Plexus
DCP	Deep Capillary Plexus
RNFL	Retinal Nerve Fiber Layer
VLDL	Very Low-Density Lipoprotein
LDL	Low-Density Lipoprotein
HDL	High-Density Lipoprotein
TG	Triglyceride
SD	Standard Deviation
bpm	Beats Per Minute
Hb	Hemoglobin
Cr	Creatinine
BUN	Blood Urea Nitrogen
AST	Aspartate Aminotransferase
ALT	Alanine Aminotransferase
GFR	Glomerular Filtration Rate
BMI	Body Mass Index
